# Inequalities of Suicide Mortality across Urban and Rural Areas: A Literature Review

**DOI:** 10.3390/ijerph19052669

**Published:** 2022-02-25

**Authors:** Judith Casant, Marco Helbich

**Affiliations:** Department of Human Geography and Spatial Planning, Faculty of Geosciences, Utrecht University, Princetonlaan 8a, 3584 CB Utrecht, The Netherlands; j.casant@students.uu.nl

**Keywords:** suicide, inequalities, urban–rural mental health, urbanicity, rurality

## Abstract

Suicide mortality is a major contributor to premature death, with geographic variation in suicide rates. Why suicide rates differ across urban and rural areas has not yet been fully established. We conducted a literature review describing the urban–rural disparities in suicide mortality. Articles were searched in five databases (EMBASE, PubMed, PsychINFO, Scopus, and Web of Science) from inception till 26 May 2021. Eligible studies were narratively analyzed in terms of the urban–rural disparities in suicides, different suicide methods, and suicide trends over time. In total, 24 articles were included in our review. Most studies were ecological and cross-sectional evidence tentatively suggests higher suicide rates in rural than in urban areas. Men were more at risk by rurality than women, but suicide is in general more prevalent among men. No obvious urban–rural pattern emerged regarding suicide means or urban–rural changes over time. Potential suicidogenic explanations include social isolation, easier access to lethal means, stigmatization toward people with mental health problems, and reduced supply of mental health services. For research progress, we urge, first, individual-level cohort and case-control studies in different sociocultural settings. Second, both rurality and urbanicity are multifaceted concepts that are inadequately captured by oversimplified typologies and require detailed assessments of the sociophysical residential environment.

## 1. Introduction

Suicide mortality is recognized worldwide as a severe public health issue. It ranks among the leading causes of global deaths and affects millions of families, communities, and individuals every year [1]. Each year, there are close to 700,000 suicide cases and even more attempts to do so [2].

The reasons why people are suicidal are complex and have a multidimensional etiology [3]. Factors at both the compositional (e.g., age and gender) and the contextual level—including biological, genetic, psychological, social, and environmental determinants—are thought to be involved [4,5,6]. The literature on mental health points to notable geographic variations in suicide mortality [7] that differ not only between but also within countries, with differences between urban and rural areas standing out [6,8,9,10,11,12,13,14,15,16,17].

An increasing number of epidemiological studies have investigated these urban–rural variations; however, the associations between urban–rural areas and suicide were not universally found and contradictory conclusions were reported [8,10,13,18,19,20]. Some studies observed significantly higher suicide rates in urban compared to rural areas, some report the opposite, while others found no difference at all. Further, it appeared that gender contributes additional heterogeneity to the results on urban–rural suicide associations. Reasons explaining these intraregional inequalities in suicide mortality are still debated and various explanations have been put forward.

On the one hand, it has been argued that the risk of suicide is considerably increased by factors such as interpersonal stress and depression [5,21,22], which can be triggered by numerous factors that differ per area, such as unemployment, work pressure, and a limited social life [23,24,25], which can eventually lead to suicidal ideation and completion. On the other hand, studies have suggested that the differences in the prevalence of mental disorders in an area, the socioeconomic circumstances therein, and the amount of mental health service utilizations available [16], physical and social isolation, access to firearms, and the stigma toward mental illness in the living environment [8,18,26], might have contributed to the urban–rural differences in suicide mortality.

Given these discrepancies between single studies, literature reviews are central to synthesize the available evidence. To date, only two systematic reviews and one narrative review (and an updated version thereof) have summarized the rich literature on the association between rurality/urbanicity and suicide [18,19,27,28]. However, the systematic review conducted by Barry and colleagues included only four English-speaking high-income countries [18], while another one focused only on elderly people in China [19]. The narrative review is selective in the material used and difficult to reproduce while lacking reliability and quality assessment of the available evidence [27]. Furthermore, the most recent narrative review was executed in 2014 [28] and new data on the urban–rural disparity in suicide mortality have been published since then. In sum, the existing reviews are fragmented geographically and/or are limited in scope, focusing on specific population groups, and thus likely rendering an incomplete picture of the urban–rural disparity in suicide worldwide.

Addressing these research needs, we conducted a review to obtain a comprehensive and up-to-date understanding of urban–rural differences in suicide mortality. Through evaluating the most recent epidemiological evidence from multiple countries narratively and assessing its quality, we sought to investigate three issues:

(1) Whether suicides occur more often in rural areas than in urban areas;
(2) Which suicide methods are used most often in urban and rural areas;
(3) What trends there are in suicides across urban and rural areas.

Given the tremendous societal burden of suicide [1] and the scientific inconclusiveness on urban–rural differences in suicide, this review is of great value as it supports place-based policy refinements to diminish health inequalities and provides an impetus for research progress by identifying conceptual and methodological shortcomings.

## 2. Materials and Methods

### 2.1. Eligibility Criteria

Only peer-reviewed journal papers were eligible for inclusion. Other literature, such as abstracts, books, dissertations, editorials, letters, opinion pieces, reports, reviews, etc., were excluded. Journal paper preprints were also excluded as they had not yet been peer-reviewed. There were no limitations on the geography or the publication date of the eligible studies. English was the language for the studies to be selected.

The study population included people of ≥15 years. The exposure of interest was living in an urban or rural area. The primary outcome of interest was suicide. This outcome may occur at any point in time following the occurrence of the exposure (i.e., living in an urban or rural area). Studies that only included people with suicidal ideation, depression, or other cognitive dysfunctions that can lead to suicidal thoughts or suicide attempts were excluded. Eligibility criteria are given in Supplementary Table S1.

### 2.2. Information Sources and Search Strategy

Our search was undertaken in five databases (EMBASE, PubMed, PsychINFO, Scopus, and Web of Science), which were searched from inception until 26 May 2021. The databases were queried for a combination of different search terms. The search query was related to “suicide(s)” AND “urban”, “rural”, “spatial” OR “geography”. The full search queries per database are presented in Supplementary Table S2.

### 2.3. Selection Process

EndNote Online was used to download, merge, and manage the reference list obtained from the five databases. Titles of the papers that were potentially relevant for this review were first screened by the first author. Duplicated records were removed. After this, studies that did not meet the inclusion criteria or that contained one of the exclusion criteria were excluded (Table S3). The remaining papers were full-text-screened by the first author to determine their eligibility for this review. Any unclear records during the title, abstract, and full-text screening were discussed by the authors until consensus was reached. Reasons for full-text exclusion are summarized in Table S3.

### 2.4. Data Collection Process

A standardized data extraction template was developed to collect the relevant data and study characteristics. The data extraction form included the following items: author, region of interest, study design, age group, suicide rates per 100,000 people for rural areas and urban areas, and rural and urban areas according to gender (if data were available according to gender).

### 2.5. Quality Assessment of Studies

The included studies underwent a critical appraisal to evaluate their quality. We used a version of the Newcastle–Ottawa scale [29] adapted for observational studies previously used in, for example, a review on elderly suicide disparities in China [19]. The eligible studies were scored by the first author on eight criteria, namely representativeness of the sample, consistency of the population in rural and urban groups, the study period, the source of data, the study controls for other variables, the assessment of the outcome, the presentation of the results, and the exploration of trends over time. Again, in the case of ambiguity in the quality assessments, records were discussed by the authors until consensus was reached.

Table S4 shows the scoring of points for these criteria. The scale’s highest score is nine points and the lowest is zero. Studies that scored 7–9 points were judged to be at low risk of bias, those that scored 5–6 points were deemed to have a moderate risk of bias, and those that scored 1–4 points were deemed to have a high risk of bias.

### 2.6. Narrative Summary

A narrative synthesis was conducted to analyze the included studies, with the risks of bias considered in concluding the state of the evidence. The narrative (1) assessed whether there were urban–rural disparities in suicide mortality in different countries worldwide, (2) described methods used for suicide and determined whether there was a difference in methods used in urban and rural areas, and (3) described trends in urban and rural suicides over time.

## 3. Results

### 3.1. Study Selection

**Figure 1:**
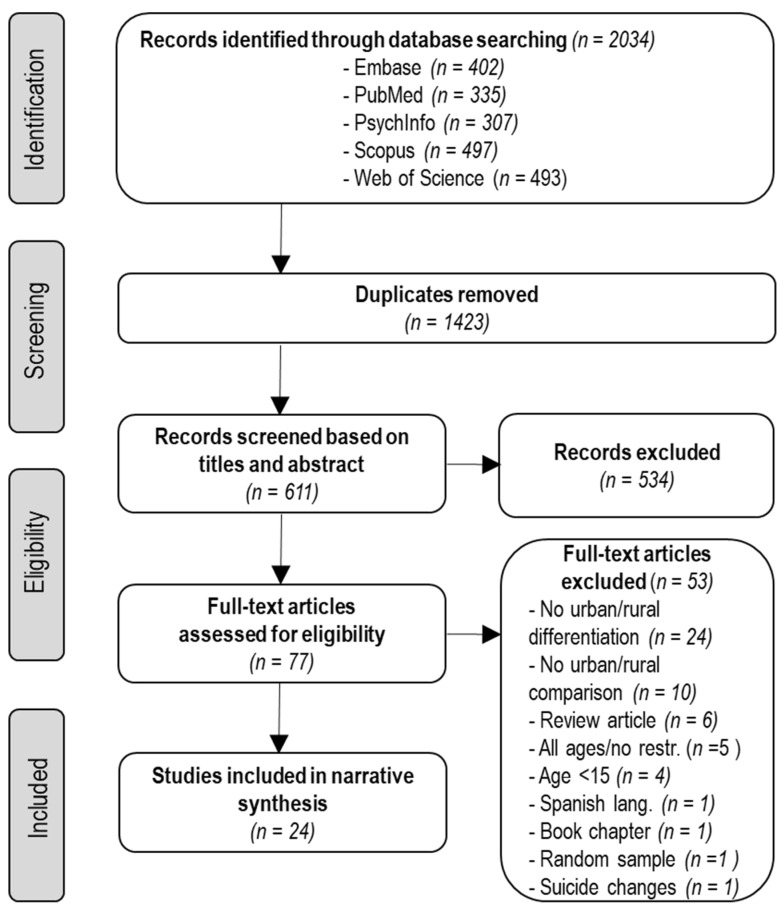
Study selection.

The electronic database search resulted in a total of 2034 studies, with 1423 duplicates. In total, the titles of 611 studies were screened to assess eligibility for inclusion and 77 studies were eligible for full-text screening. Out of these 77 studies, 53 papers were excluded (Table S3). The reasons for exclusion are summarized in Figure 1. In total, 24 articles met our inclusion criteria for narrative synthesis.

### 3.2. Study Characteristics

The characteristics of the included studies are shown in Table 1. The majority used data of a cross-sectional nature [30,31,32,33,34]. Because the suicide rates were usually based on administrative databases (e.g., death registers) covering the entire population, and given that privacy needed to be guaranteed, ecological designs were often applied [35,36]; other designs were scarce (e.g., cohort design [37]). The year of publication of the included articles covered the period 1977–2020. The analyzed data therein covered the period 1967–2018. The studies were from numerous countries, with most from Australia (*n* = 9) and the United States (U.S.) (*n* = 3). Only five studies were executed in non-high-income countries. The majority of articles stratified the analyses by gender (Table 1), except one, which just included suicide rates for men [38].

### 3.3. Differences in Suicide Rates between Urban and Rural Areas

Multiple studies reported that although rural suicide rates among males exceeded those among males in urban areas, there seems to be no urban–rural difference in female suicides [16,31,40,43,48]. Saunderson et al. found the same for England and Wales; however, data for 1989–92 revealed that female suicide rates did show an excess in the most densely populated areas [36]. In Scotland, there were higher rural suicide completion rates for men in the 1980s and 1990s, but rurality showed a diminished effect among female suicides [13]. These results contradicted a Japanese study that compared a rural with an urban area, whereas females were more affected by rurality and—independent of gender—the rural area had pronounced suicide rates [50]. Data for South Korea showed higher rural suicide completion rates in 2005 for both men and women [45].

The majority of suicides among Australian public mental health service patients were rural [47], which is in line with Chang et al., who reported higher, though insignificant, suicide rates per 100,000 people in rural versus urban Taiwan [35]. In the Belarus countryside higher suicide rates were reported [46].

Carriere et al. assessed U.S. data for 2002–2016 and found more female urban suicides than female rural ones [42]. Both Page et al. for young Australian adults (1979–2003) [33] and Yip et al. for Australia and Beijing (China) (1991–1996) [17] reported higher urban female suicide rates, while suicide rates were higher among rural Australian men. Only one study—from New Zealand (1980–1982)—established a significantly higher suicide rate in urban areas for both sexes [34].

Pesonen et al. focused on Finnish males in the 1980s and 1990s, but could not confirm significant geographic inequalities in suicide mortality for eastern Finland (though rates for the countryside were higher) with regionally diverging rates (e.g., a decline in urban areas) [38]. Congruent with suicide rates (2003–2017) for U.S. Veterans Affairs patients [37], no significant differences were observable in Australian urban and rural suicide rates (1986–1990) [41]. Others did not find urban–rural differences [30,44,49,51].

### 3.4. Methods of Suicide

About half of the 24 studies distinguished between suicide means across urban and rural areas (Table 1). Common means, in general, included hanging, poisoning, firearms, drowning, and jumping. Others, such as medical overdose, cutting, or suffocation were infrequently mentioned [13,16,32,38,49,50].

In rural Finland, violent suicide methods (e.g., firearms, jumping) were more prevalent than nonviolent ones (e.g., poisoning) [38]. Firearm deaths were more common among men and women living in rural areas of the U.S. [32] and Australia [16], though it was also a prevalent means in American cities [37]. The Australian results were contrasted by Sankaranarayanan et al. [47], who found that hanging was widespread among mental health service patients in 2003–2007; firearms ranked second for rural suicides. Though jumping was the second most common suicide means in urban areas, it seldom occurred in the countryside [47]. Elsewhere (e.g., Japan, Taiwan) hanging remained prevalent [35,50], while in rural South Korea people often chose poisoning by pesticides [45]. Suicide data for Shandong, China, between 1991 and 2010 showed a considerable increase in poisoning by pesticides in urban areas, as well as an increase in hanging for rural areas [49].

### 3.5. Trends over Time

Temporal trends were less often analyzed. Between the mid-1980s and the mid-1990s, the male rural suicide rates in Ireland rose by 50%, while male urban suicide rates remained stable [43]. For men and women in urban and rural areas, suicide rates increased between 1981 and 1999 in Scotland [13]; results were similar for Taiwan (1991 to 2007) [30] but were only partially in line with those for Australia [33]. Therein, urban and rural male suicide rates increased between 1979 and 1998; no significant change was observed for females in urban or rural areas. U.S. data covering three decades (1970–1997) indicated that urban–rural differences also increased for male suicides [48].

For Belarus between 1990 and 2005, female suicide rates in rural areas increased substantially, while urban female suicide rates decreased; those for urban and rural men remained steady [46]. Likewise, after a decrease in urban and rural Finland between 1988 and 1993, rural male suicide rates increased, while those in urban areas declined between 1993 and 1997 [38]. These trends were not confirmed in Shandong (China) between 1991 and 2010, with an overall significant decrease in suicide rates and a stronger rural decline, which was more evident among females [49]. There were significantly more urban suicide completions in the 1980s in New Zealand, but by the 1990s, this difference disappeared [34]. Trends of suicide rates were stable in Australia and Beijing in 1991–1996 for both urban and rural areas [17].

### 3.6. Risk of Bias

The summary of the quality assessment including detailed ratings of the critical appraisal is presented in Table S5. Overall, of the 24 studies, 21 were classified as low risk of bias, five as moderate risk of bias, and one as high risk of bias. Studies frequently did not assess trends over time, reported the results incompletely, and had limited covariate adjustments. Two articles had the highest score of nine points [13,49], nine scored eight points [16,32,33,34,35,37,39,46,48], ten scored seven points [17,30,31,36,38,40,41,42,43,44], two scored six points [45,47], one scored five points [50].

## 4. Discussion

### 4.1. Principal Findings

Decades of research on suicide mortality across urban and rural areas have yielded numerous major findings. The overall results from the 24 studies included in this literature review provided indicative evidence of inequalities in suicide completions across urban and rural areas, whereby suicide is more prevalent in the countryside. In contrast to mental health studies, which frequently attribute higher risk to urban living [7], our results suggest a reversed pattern for suicide mortality; evidence concerning pronounced suicide risk in cities was limited [34]. Although most studies were conducted in Australia or the U.S., urban–rural disparity in suicides appeared worldwide and was not limited to high-income countries.

Further, it was apparent that in rural areas, men are more at risk than women, which is congruent with the notion that suicide mortality is generally more prevalent among men [5]. Suicide methods also varied across urban and rural areas. There is some indication that firearms and poisoning by pesticides are frequent suicide means in rural areas, while no specific pattern was observable for urban areas. Given the reviewed evidence, we cannot draw a definite conclusion about urban–rural suicide temporal trends, because the reviewed studies covered long observation periods in different countries.

### 4.2. Possible Explanation and Interpretation of the Findings

At least four potential suicidogenic risk factors have been proposed in the literature to explain urban–rural inequalities in death by suicide.

#### 4.2.1. Social Integration

People in the countryside are possibly stigmatized when they talk about their mental health problems [43], while the communication of suicidal ideations is difficult in anonymous social conditions [36]. This corresponds with the Durkheimian theory [52] that low levels of social integration are associated with high suicide rates [48]. People in urban areas have thus greater potential for social integration and are therefore at lower risk [53]. Moreover, some population groups, such as married people, are characterized by greater social integration than others (e.g., divorced people) [54]. This explanation corresponds with empirical evidence, namely that divorced, separated, widowed, or persons who were never married are at higher risk of suicide mortality [20,39]. Other groups with less social integration and more isolation, such as the elderly [55], or those less engaged in religious practices [56], are also prone to suicide [50].

#### 4.2.2. Socioeconomic Risk Factors

Suicide mortality is heightened not only by a lack of social integration, but also by unemployment and higher levels of poverty [42,57]. Suicide rates are higher for unemployed people and those with a lower socioeconomic status (i.e., people with a lower education and a lower income) [39,58]. It was proposed that less favorable socioeconomic standards on an individual-level (e.g., limited economic resources) and area-level deprivation [59,60] in rural areas can explain the urban–rural differences [17,46].

#### 4.2.3. Availability of Mental Health Services

Another possible reason for urban–rural suicide disparities is related to the availability and quality of mental healthcare services (e.g., general practitioners, psychiatrists) [28,61]. People who live in rural areas may be less likely to seek help due to longer travel distances to medical care facilities. Receiving no or low-quality mental health treatment increases the chance of suicide [18], as mental illness is a serious risk factor [62].

#### 4.2.4. Availability of Suicide Methods

Certain suicide methods are more available in rural areas, possibly stimulating suicide thoughts [63]. For example, firearms are widespread in rural areas and since firearms have a high case-fatality rate, suicide attempts almost always succeed [64,65]. Easy access to pesticides, which are often highly toxic [66], makes self-poisoning common in rural areas [67]. Rural and remote areas, where a higher proportion of the population work in agriculture and has eased access to pesticides, also tend to have a higher pesticide suicide rate [33,35].

### 4.3. Strengths and Limitations

Our review extends previous reviews by including the most recent evidence on urban–rural differences in suicide mortality. Based on our narrative synthesis, we provided insights into urban–rural disparities and possible underlying suicidogenic mechanisms. The methodological weaknesses of our review are the possible elimination of relevant studies due to our inclusion criteria, the absence of a systematic assessment of the literature, and because we excluded studies written in a language other than English [68]. We did an initial title-based study selection, which likely missed some relevant studies and excluded those that only controlled for urban–rural differences as confounding factors, without explicitly mentioning this fact in the title. Due to a large amount of heterogeneity across studies (e.g., study design, study period, and location), we did not perform meta-analyses, which would provide a statistically grounded assessment of the urban–rural suicide associations. Another limitation is that only the first author reviewed the studies, which may have introduced a selection bias [69]. However, any possible assessment bias resulting from that seems negligible because ambiguities were virtually nonexistent. Most evidence stems from European and Anglo-Saxon countries, and thus our summary might suffer from a cultural bias. Finally, the comparability of the reviewed evidence was hampered due to the various ways in which urban–rural areas were operationalized and the extensive reviewing period. It is likely that urbanization processes that are constantly shaping both urban and rural areas were at play and impede rigorous comparisons of urban and rural suicide rates over our extensive study period.

### 4.4. Implications for Public Health Policy and Practice

Because suicide is a preventable cause of death, more attention should be paid to spatially targeted suicide prevention rather than universally applicable “one-size-fits-all” prevention policies. For example, suicide rates remain higher in rural areas than in urban areas in almost all countries, and prevention must be reinforced especially for men. Ongoing urbanization worldwide, with its rural–urban migration flows, may have implications for the demographic profile of rural and urban areas and, in turn, may alter suicide patterns. Furthermore, urbanization can also affect mental health [7], which is commonly believed to be related to suicide risk [62]. More attention should be paid to the design and implementation of suicide prevention programs targeting the mechanisms thought to put rural people at risk. These mechanisms include less favorable socioeconomic factors, easier access to firearms and poisons, limited access to health services, and social isolation.

### 4.5. Future Research Priorities

To advance our understanding of the geographic variation in suicide, there are at least four conceptual and methodological implications for further research. First, it is recommended that future studies examine urban–rural disparity by age [6] and socioeconomic factors [20], because the disparity between urban and rural suicides has long been noted and both age and socioeconomic factors are additional confounders that influence the urban–rural disparity in suicides.

Second, with only a few exceptions [37,39], studies have largely been ecological, using data aggregated to administrative units. The statistical results are likely to be not only severely confounded due to a lack of individual-level data, which can result in spurious urban–rural suicide associations, but also prone to ecological fallacy [70] and the modifiable areal unit problem [71]—namely, that analyses are vulnerable to changes in the level of aggregation and zoning. Further, these research design aspects make it difficult to establish causality between urban/rural areas and suicide mortality. Cautious communication to the public is essential to ensure a proper understanding of the results and any limitations in the data. Progress can be achieved through research designs at the individual level, including case-control [72] and cohort studies [73]. Relatedly, future studies also need to acknowledge that people move home over their life course [74]. To exclude spurious associations between suicide and urban/rural areas due to selective migration [75], it seems vital to assess how environmental changes (e.g., due to a relocation from urban to rural areas) relate to suicide mortality.

Third, comparative results must be interpreted with caution because of methodological variations in representing urban and rural areas. Due to a lack of agreement on how to best operationalize rurality, reported results are likely to depend to some degree on the way in which urban and rural areas are geographically delineated. The approaches used range from binary urban–rural strata to fine-grained stratifications that are intended to represent the continuum between cities and the countryside [10]. Complexity is further increased by the diverse threshold values that are used to define what is urban/rural (e.g., number of people per area). To homogenize studies by increasing their comparability, one important direction is to use a more coherent way of defining rurality/urbanicity. Here, a classification developed by the Organisation for Economic Co-operation and Development seems promising [76].

Fourth, we question whether urban–rural differences can be captured meaningfully with a single proxy variable that may be a poor substitute for area-level compositional effects (e.g., air pollution [77], green space [57], deprivation [59,60]). Rather than using a single overly crude urban–rural indicator, future research is advised to distinguish between environmental and social characteristics to add in-depth meaning to urban and rural geographies. Methodological rigor in terms of refined exposure assessments can also be achieved by the application of small-scale administrative areas within which people live or even their residential address location [57].

## 5. Conclusions

Although vast and long-running epidemiological studies have investigated geographic variation in urban–rural suicide completion rates, their results are not consistent. We therefore carried out a narrative review to provide an up-to-date overview of the literature and emphasize future research priorities. In general, the findings of the reviewed studies (1) tend toward higher suicide completion rates in rural areas vs. urban areas, (2) revealed that especially men seem to be affected by rurality, and (3) were inconclusive concerning an urban–rural pattern in suicide means and temporal trends. Suicidogenic mechanisms that possibly play a role in putting rural people at risk include social isolation and less intimate face-to-face contact with family and friends, easier access to lethal means, stigmatization toward mental health problems, and reduced supply of mental health services. Gaps in the body of evidence suggest a need for scientific rigor in terms of individual-level longitudinal studies in which rurality is represented by detailed-scaled multivariable measures of the sociophysical residential environment.

## Figures and Tables

**Table 1:** Characteristics of the eligible studies.

Study	Population	Region	Research Design	Indicator	*N*	Study Period	Age Group	Suicide Rate (per 100,000 People)					
								Rural	Urban	Rural Women	Urban Women	Rural Men	Urban Men
[39] *	All	Canada	Cohort	Suicide rates	2,735,152	1991–2001	≥25	-	-	7.5	6.0	31.4	18.6
[40]	All	Australia	Ecological	Suicide rates	6 states	1997–2000	≥20	-	-	5.7	5.6	24.0	20.2
[41] *	All	Queensland, Australia	Ecological	Suicide rates	1 state	1986–1990	15–19	-	-	6.5	1.7	24.1	18.9
							20–29	-		4.2	10.2	29.6	33.2
[42] **	All	United States	Ecological	County mortality rates	50 states	2002	≥20	17.2	13.2	-	-	-	-
						2016		23.2	15.8	-	-	-	-
[30]	All	Taiwan	Ecological	Suicide rates	358 districts	1999–2001	≥15	-	-	14.3	11.1	31.4	21.3
						2002–2004		-	-	15.1	12.8	30.4	27.2
						2005–2007		-	-	17.3	15.1	37.8	31.5
[35] *	All	Taiwan	Ecological	Suicide rates	358 districts	2002–2009	≥15	26.3	21.2	-	-	-	-
[31]	All	Australia	Ecological	Suicide rates	6 states	2004–2008	15–19	-	-	4.56	4.60	18.19	15.67
[43] *,**	All	Ireland	Ecological	Suicide rates	27 counties	1976	≥15	-	-	3.5	4.5	8.0	7.5
						1993		-	-	4.5	4.5	20.0	15.5
[13] *,**	All	Australia	Ecological	Suicide rates	32 council areas	1981–1984	15–39	-	-	8.8	7.5	29.0	20.0
						1997–1999		-	-	13.2	10.5	45.0	36.5
[32]	Veterans Affairs users	United States	Ecological	Suicide rates	50 states	2004–2005	≥18	38.76	31.45	-	-	-	-
						2007–2008		39.62	32.44	-	-	-	-
[44] *	Migrants	New South Wales, Australia	Ecological	Suicide rates	1 state	1985–1994	≥15	-	-	7.9	6.5	38.2	19.9
[33] *	All	Australia	Ecological	Suicide rates	6 states	1979–1983		-	-	5.3	8.6	19.2	19.5
						1984–1988		-	-	6.3	7.6	23.7	21.8
						1989–1993		-	-	5.4	7.2	22.9	22.9
						1994–1998		-	-	5.9	7.1	24.2	24.2
						1999–2003		-	-	6.1	6.8	22.4	22.4
[45]	All	South Korea	Ecological	Suicide rates	9 provinces	2005	≥15	35.4	16.3	26.1	20.0	63.3	40.3
[34] *,**	All	New Zealand	Ecological	Suicide rates	10 provinces	1981	≥15	-	-	6.0	4.0	11.8	14.6
						2000		-	-	5.3	5.5	21.0	19.5
[38] *	Males	Eastern Finland	Ecological	Suicide rates	1 province	1988	≥15	-	-	-	-	84.0	53.0
						1992		-	-	-	-	53.0	52.0
						1997		-	-	-	-	80.0	36.0
[46] *	All	Belarus	Ecological	Suicide rates	6 oblasts	1990	≥15	-	-	8.69	7.74	50.48	31.96
						1995		-	-	10.02	9.48	80.08	52.27
						2000		-	-	11.73	8.58	99.68	50.33
						2005		-	-	11.67	6.78	94.73	38.79
[47] *	Mental Health Service	Australia	Ecological	Suicide rates	580	2003–2007	≥15	3.1	1.1	-	-	-	-
[36]	All	England and Wales	Ecological	Suicide rates	311 districts	1989–1992	≥15	-	-	-	-	-	-
[37] *	Veterans Affairs users	United States	Cohort	Suicide rates	6,120,355	2003–2005	≥18	30.6	27.2	13.1	9.6	31.6	29.0
						2006–2008		32.5	27.8	8.6	9.6	34.0	29.7
						2009–2011		33.2	29.1	13.8	13.1	34.4	30.9
						2012–2014		34.8	30.3	18.1	11.9	35.9	32.5
						2015–2017		35.6	31.0	16.8	12.4	37.0	33.3
[48] *	All	Canada	Ecological	Suicide rates	3103 counties	1970–1974	≥15	-	-	4.13	8.70	20.71	19.84
						1975–1979		-	-	5.31	7.61	21.31	20.36
						1980–1984		-	-	4.58	6.00	34.24	19.17
						1985–1989		-	-	4.43	5.25	24.57	19.58
						1990–1994		-	-	4.14	4.57	25.73	18.74
						1995–1997		-	-	4.01	4.05	26.88	17.45
[49] *	All	Shandong, China	Ecological	Suicide rates	19 counties	1991–1995	≥15	40.02	6.62	40.75	6.88	39.26	6.58
						1996–2000		31.16	7.71	30.28	6.87	32.04	8.82
						2001–2005		21.24	4.95	18.99	3.97	23.55	5.87
						2006–2010		19.00	5.32	17.33	4.73	20.65	5.90
[16] *	All	Australia	Ecological	Suicide rates	6 states	1996–1998	≥20	-	-	7.3	7.2	34.7	28.2
[50]	All	Japan	Ecological	Suicide rates	47 prefectures	1988	≥65	240.4	38.3	277.3	26.5	185.0	53.9
[17]	All	Australia	Ecological	Suicide rates	1 country	1991–1996	≥15	-	-	5.4	6.7	30.5	25.7
[17]	All	Beijing	Ecological	Suicide rates	1 city	1991–1996	≥15	-	-	17.1	6.0	16.3	5.7

* Age-adjusted rates; ** data collected from figures. Studies used different ways to standardize suicide rates per 100,000 people, which challenges an exact comparison across the studies. The provided urban–rural taxonomy tries to harmonize multiple approaches on how urban and rural areas were operationalized across the reviewed studies ranging from a binary classification to an urban–rural continuum.

## Data Availability

Not applicable.

## Supplementary Materials

The following are available online at https://www.mdpi.com/article/10.3390/ijerph19052669/s1. Table S1: Eligibility criteria, Table S2: Search strategy for each database, Table S3: Exclusion reasons for full-text articles, Table S4: Criteria for quality assessment of the selected studies, and Table S5: Newcastle–Ottawa risk of bias scores for the included studies.

## References

[1] Naghavi M. (2019). Global, regional, and national burden of suicide mortality 1990 to 2016: Systematic analysis for the Global Burden of Disease Study 2016. BMJ.

[2] World Health Organization (2021). Suicide Worldwide in 2019: Global Health Estimates.

[3] Diaz-Oliván I., Porras-Segovia A., Barrigón M.L., Jiménez-Muñoz L., Baca-Garcia E. (2021). Theoretical models of suicidal behaviour: A systematic review and narrative synthesis. Eur. J. Psychiatry.

[4] Li Z., Page A., Martin G., Taylor R. (2011). Attributable risk of psychiatric and socio-economic factors for suicide from individual-level, population-based studies: A systematic review. Soc. Sci. Med..

[5] Turecki G., Brent D.A. (2016). Suicide and suicidal behaviour. Lancet.

[6] Forte A., Vichi M., Ghirini S., Orri M., Pompili M. (2021). Trends and ecological results in suicides among Italian youth aged 10–25 years: A nationwide register study. J. Affect. Disord..

[7] Ventriglio A., Torales J., Castaldelli-Maia J.M., De Berardis D., Bhugra D. (2021). Urbanization and emerging mental health issues. CNS Spectr..

[8] Fontanella C.A., Hiance-Steelesmith D.L., Phillips G.S., Bridge J.A., Lester N., Sweeney H.A., Campo J.V. (2015). Widening rural-urban disparities in youth suicides, United States, 1996–2010. JAMA Pediatr..

[9] Yoshioka E., Hanley S.J.B., Sato Y., Saijo Y. (2021). Geography of suicide in Japan: Spatial patterning and rural–urban differences. Soc. Psychiatry Psychiatr. Epidemiol..

[10] Helbich M., Blüml V., de Jong T., Plener P.L., Kwan M.-P., Kapusta N.D. (2017). Urban-rural inequalities in suicide mortality: A comparison of urbanicity indicators. Int. J. Health Geogr..

[11] Kanamori M., Kondo N., Juárez S.P., Cederström A., Stickley A., Rostila M. (2021). Does increased migration affect the rural-urban divide in suicide? A register-based repeated cohort study in Sweden from 1991 to 2015. Popul. Space Place.

[12] Kapusta N.D., Zorman A., Etzersdorfer E., Ponocny-Seliger E., Jandl-Jager E., Sonneck G. (2008). Rural-urban differences in Austrian suicides. Soc. Psychiatry Psychiatr. Epidemiol..

[13] Levin K.A., Leyland A.H. (2005). Urban/rural inequalities in suicide in Scotland, 1981–1999. Soc. Sci. Med..

[14] Ma X., Xiang Y.-T., Cai Z.-J., Li S.-R., Xiang Y.-Q., Guo H.-L., Hou Y.-Z., Li Z.-B., Li Z.-J., Tao Y.-F. (2009). Lifetime prevalence of suicidal ideation, suicide plans and attempts in rural and urban regions of Beijing, China. Aust. N. Z. J. Psychiatry.

[15] Middleton N., Sterne J.A., Gunnell D. (2006). The geography of despair among 15-44-year-old men in England and Wales: Putting suicide on the map. J. Epidemiol. Community Health.

[16] Taylor R., Page A., Morrell S., Harrison J., Carter G. (2005). Social and psychiatric influences on urban-rural differentials in Australian suicide. Suicide Life-Threat. Behav..

[17] Yip P.S.F., Callanan C., Yuen H.P. (2000). Urban/rural and gender differentials in suicide rates: East and West. J. Affect. Disord..

[18] Barry R., Rehm J., de Oliveira C., Gozdyra P., Kurdyak P. (2020). Rurality and risk of suicide attempts and death by suicide among people living in four English-speaking high-income countries: A systematic review and meta-analysis. Can. J. Psychiatry.

[19] Li M.Z., Katikireddi S.V. (2019). Urban-rural inequalities in suicide among elderly people in China: A systematic review and meta-analysis. Int. J. Equity Health.

[20] Hagedoorn P., Groenewegen P.P., Roberts H., Helbich M. (2020). Is suicide mortality associated with neighbourhood social fragmentation and deprivation? A Dutch register-based case-control study using individualised neighbourhoods. J. Epidemiol. Community Health.

[21] Robbins D.R., Alessi N.E. (1985). Depressive symptoms and suicidal behavior in adolescents. Am. J. Psychiatry.

[22] Roy A., Breier A., Doran A.R., Pickar D. (1985). Life events in depression. Relationship to subtypes. J. Affect. Disord..

[23] Fusé T. (1980). Suicide and culture in Japan: A study of seppuku as an institutionalized form of suicide. Soc. Psychiatry.

[24] Koo J., Cox W.M. (2008). An economic interpretation of suicide cycles in Japan. Contemp. Econ. Policy.

[25] Kuroki M. (2010). Suicide and unemployment in Japan: Evidence from municipal level suicide rates and age-specific suicide rates. J. Soc. Econ..

[26] Searles V.B., Valley M.A., Hedegaard H., Betz M.E. (2014). Suicides in urban and rural counties in the United States, 2006–2008. Crisis.

[27] Hirsch J.K. (2006). A review of the literature on rural suicide: Risk and protective factors, incidence, and prevention. Crisis J. Crisis Interv. Suicide Prev..

[28] Hirsch J.K., Cukrowicz K.C. (2014). Suicide in rural areas: An updated review of the literature. J. Rural Ment. Health.

[29] Wells G., Shea B., O’Connell D., Robertson J., Peterson J., Welch V., Losos M., Tugwell P. The Newcastle-Ottawa Scale (NOS) for assessing the quality of nonrandomized studies in meta-analysis. http://www.ohri.ca/programs/clinical_epidemiology/oxford.asp.

[30] Chang S.S., Gunnell D., Wheeler B.W., Yip P., Sterne J.A.C. (2010). The evolution of the epidemic of charcoal-burning suicide in Taiwan: A spatial and temporal analysis. PLoS Med..

[31] Cheung Y.T.D., Spittal M.J., Pirkis J., Yip P.S.F. (2012). Spatial analysis of suicide mortality in Australia: Investigation of metropolitan-rural-remote differentials of suicide risk across states/territories. Soc. Sci. Med..

[32] McCarthy J.F., Blow F.C., Ignacio R.V., Ilgen M.A., Austin K.L., Valenstein M. (2012). Suicide among patients in the Veterans Affairs Health System: Rural-urban differences in rates, risks, and methods. Am. J. Public Health.

[33] Page A., Morrell S., Taylor R., Dudley M., Carter G. (2007). Further increases in rural suicide in young Australian adults: Secular trends, 1979–2003. Soc. Sci. Med..

[34] Pearce J., Barnett R., Jones I. (2007). Have urban/rural inequalities in suicide in New Zealand grown during the period 1980–2001?. Soc. Sci. Med..

[35] Chang S.S., Lu T.H., Sterne J.A.C., Eddleston M., Lin J.J., Gunnell D. (2012). The impact of pesticide suicide on the geographic distribution of suicide in Taiwan: A spatial analysis. BMC Public Health.

[36] Saunderson T., Haynes R., Langford I.H. (1998). Urban-rural variations in suicides and undetermined deaths in England And Wales. J. Public Health.

[37] Shiner B., Peltzman T., Cornelius S.L., Gui J., Forehand J., Watts B.V. (2020). Recent trends in the rural–urban suicide disparity among veterans using VA health care. J. Behav. Med..

[38] Pesonen T.M., Hintikka J., Karkola K.O., Saarinen P.I., Antikainen M., Lehtonen J. (2001). Male suicide mortality in eastern Finland—Urban-rural changes during a 10-year period between 1988 and 1997. Scand. J. Public Health.

[39] Burrows S., Auger N., Gamache P., Hamel D. (2013). Leading causes of unintentional injury and suicide mortality in Canadian adults across the urban-rural continuum. Public Health Rep..

[40] Caldwell T.M., Jorm A.F., Dear K.B.G. (2004). Suicide and mental health in rural, remote and metropolitan areas in Australia. Med. J. Aust..

[41] Cantor C.H., Coory M. (1993). Is there a rural suicide problem?. Aust. J. Public Health.

[42] Carriere D.E., Marshall M.I., Binkley J.K. (2019). Response to economic shock: The impact of recession on rural-urban suicides in the United States. J. Rural Health.

[43] Kelleher M.J., Corcoran P., Keeley H.S., Chambers D., Williamson E., McAuliffe C., Burke U., Byrne S. (2002). Differences in Irish urban and rural suicide rates, 1976–1994. Arch. Suicide Res..

[44] Morrell S., Taylor R., Slaytor E., Ford P. (1999). Urban and rural suicide differentials in migrants and the Australian-born, New South Wales, Australia 1985–1994. Soc. Sci. Med..

[45] Park B., Lester D. (2012). Rural and urban suicide in South Korea. Psychol. Rep..

[46] Razvodovsky Y., Stickley A. (2009). Suicide in urban and rural regions of Belarus, 1990–2005. Public Health.

[47] Sankaranarayanan A., Carter G., Lewin T. (2010). Rural-urban differences in suicide rates for current patients of a public mental health service in Australia. Suicide Life-Threat. Behav..

[48] Singh G.K., Siahpush M. (2002). Increasing rural-urban gradients in US suicide mortality, 1970–1997. Am. J. Public Health.

[49] Sun J., Guo X., Zhang J., Jia C., Xu A. (2013). Suicide rates in Shandong, China, 1991–2010: Rapid decrease in rural rates and steady increase in male-female ratio. J. Affect. Disord..

[50] Watanabe N., Hasegawa K., Yoshinaga Y. (1995). Suicide in later life in Japan: Urban and rural differences. Int. Psychogeriatr..

[51] Burrows S., Laflamme L., Vaez M. (2007). Sex-specific suicide mortality in the South African urban context: The role of age, race, and geographical location. Scand. J. Public Health.

[52] Durkheim É. (1951). Suicide: A Study in Sociology.

[53] Faupel C.E., Kowalski G.S., Starr P.D. (1987). Sociology one law, religion and suicide in the urban context. J. Sci. Study Relig..

[54] Masumura W.T. (1977). Social integration and suicide: A test of Durkheim’s theory. Behav. Sci. Res..

[55] Heuser C., Howe J. (2018). The relation between social isolation and increasing suicide rates in the elderly. Qual. Ageing Older Adults.

[56] Duberstein P.R., Conwell Y., Conner K.R., Eberly S., Evinger J.S., Caine E.D. (2004). Poor social integration and suicide: Fact or artifact? A case-control study. Psychol. Med..

[57] Helbich M., O’Connor R.C., Nieuwenhuijsen M., Hagedoorn P. (2020). Greenery exposure and suicide mortality later in life: A longitudinal register-based case-control study. Environ. Int..

[58] Helbich M., Plener P.L., Hartung S., Blüml V. (2017). Spatiotemporal suicide risk in Germany: A longitudinal study 2007–11. Sci. Rep..

[59] Rehkopf D.H., Buka S.L. (2006). The association between suicide and the socio-economic characteristics of geographical areas: A systematic review. Psychol. Med..

[60] Cairns J.-M., Graham E., Bambra C. (2017). Area-level socioeconomic disadvantage and suicidal behaviour in Europe: A systematic review. Soc. Sci. Med..

[61] Kapusta N.D., Posch M., Niederkrotenthaler T., Fischer-Kern M., Etzersdorfer E., Sonneck G. (2010). Availability of mental health service providers and suicide rates in Austria: A nationwide study. Psychiatr. Serv..

[62] Chesney E., Goodwin G.M., Fazel S. (2014). Risks of all-cause and suicide mortality in mental disorders: A meta-review. World Psychiatry.

[63] Judd F., Cooper A.-M., Fraser C., Davis J. (2006). Rural suicide—People or place effects?. Aust. N. Z. J. Psychiatry.

[64] Burnley I.H. (1995). Socioeconomic and spatial differentials in mortality and means of committing suicide in New South Wales, Australia, 1985–1991. Soc. Sci. Med..

[65] Pettrone K., Curtin S.C. (2020). Urban-Rural Differences in Suicide Rates, by Sex and Three Leading Methods: United States, 2000–2018.

[66] Kong Y., Zhang J. (2010). Access to farming pesticides and risk for suicide in Chinese rural young people. Psychiatry Res..

[67] Cha E.S., Khang Y.-H., Lee W.J. (2014). Mortality from and incidence of pesticide poisoning in South Korea: Findings from national death and health utilization data between 2006 and 2010. PLoS ONE.

[68] Chaparro-Narváez P., Díaz-Jiménez D., Castañeda-Orjuela C. (2019). The trend in mortality due to suicide in urban and rural areas of Colombia, 1979–2014. Biomedica.

[69] Waffenschmidt S., Knelangen M., Sieben W., Bühn S., Pieper D. (2019). Single screening versus conventional double screening for study selection in systematic reviews: A methodological systematic review. BMC Med. Res. Methodol..

[70] Robinson W.S. (2009). Ecological correlations and the behavior of individuals. Int. J. Epidemiol..

[71] Openshaw S. (1981). The modifiable areal unit problem. Quant. Geogr. A Br. View.

[72] Hagedoorn P., Helbich M. (2021). Longitudinal exposure assessments of neighbourhood effects in health research: What can be learned from people’s residential histories?. Health Place.

[73] Hagedoorn P., Helbich M. (2022). Longitudinal effects of physical and social neighbourhood change on suicide mortality: A full population cohort study among movers and non-movers in the Netherlands. Soc. Sci. Med..

[74] Helbich M. (2018). Toward dynamic urban environmental exposure assessments in mental health research. Environ. Res..

[75] Jokela M. (2014). Are neighborhood health associations causal? A 10-year prospective cohort study with repeated measurements. Am. J. Epidemiol..

[76] OECD Functional Urban Areas by Country. http://www.oecd.org/cfe/regional-policy/functionalurbanareasbycountry.htm.

[77] Davoudi M., Barjasteh-Askari F., Amini H., Lester D., Mahvi A.H., Ghavami V., Ghalhari M.R. (2021). Association of suicide with short-term exposure to air pollution at different lag times: A systematic review and meta-analysis. Sci. Total Environ..

