# Supplementary material for: Inequalities of Suicide Mortality across Urban and Rural Areas: A Literature Review
Source: Int J Environ Res Public Health. 2022 Feb 25;19(5):2669. doi: 10.3390/ijerph19052669 (PMC8909802; doi:10.3390/ijerph19052669)
Supplement: Supplementary file 1 [file ijerph-19-02669-s001.zip › ijerph-1587441-supplementary.pdf]

Supplementary Materials

Table S1: Eligibility criteria

Category	Inclusion	Exclusion
Population	Studies on people ≥15 years	Studies that focus on children or adolescents (≤15 years); studies that did not specify the age of the population; studies without age restriction of the population; animal studies.
Exposure	Urban or rural (living) area or environment.	No measurement of urban or rural (living) area of environment; no clear description of how urban or rural area is defined; no comparison between urban and rural areas; no use of the words urban or rural; areas or cities used that were not defined as urban or rural.
Outcome	Completed suicide.	Suicidal ideation, suicide attempt(s), depression, anxiety, stress, other cognitive dysfunctions that can affect suicidal thoughts.

Table S2: Search strategy for each database (May 26, 2021)

Database	Search strategy	Number of studies
Embase	suicide:ti AND (rural:ti OR urban:ti OR spatial:ti OR geography:ti) AND [english]/lim	402
PubMed	("suicide"[Title] AND ("urban"[Title] OR "rural"[Title] OR "geography"[Title]) AND (english[Filter])	335
PsychInfo	#1 (suicide and (urban or rural or spatial or geography)).ti.<br>#2 limit 1 to English language	307
Scopus	#1 (TITLE (suicide) AND LANGUAGE (english))<br><br>#2 (TITLE (urban OR rural OR spatial OR geography) AND LANGUAGE (english))<br><br>#1 AND #2	497
Web of Science	TITLE: (suicide AND (urban OR rural OR spatial OR geography)) AND LANGUAGE: (English)	493

Table S3: Exclusion reasons for full-text articles

Reasons	Reference
No definition of rural or urban areas *(n=24)*	Asevedo et al. [1]; Burnley [2]; Burrows et al. [3]; Chambers & Harvey [4]; Chang et al. [5]; Congdon [6]; Dantas et al. [7]; Dudley et al. [8]; Günay & Mert Kantar [9]; Gunnell et al. [10]; Guo et al. [11]; Heleniak [12]; Kelly et al. [13]; Lin et al. [14]; Macente & Zandonade [15]; Ngamini Ngui et al. [16]; Phillips [17]; Qi et al. [18]; Santana et al. [19]; Stark et al. [20]; Sugg et al. [21]; Tomita et al. [22]; Wilkinson & Israel [23]; Yoshioka et al. [24]
No comparison of rural and urban suicide rates *(n=10)*	Cullen & Connolly [25]; Duckworth & McBride [26]; Gallagher & Sheehy [27]; Gelfand [28]; Kim & Kim [29]; Nestadt et al. [30]; Qi et al. [31]; Renwick et al. [32]; Searles et al. [33]; Tamosiunas et al. [34]
Review article *(n=6)*	Baume & Clinton [35]; Crnek-Georgeson et al. [36]; Hirsch [37]; Hirsch & Cukrowicz [38]; Lester [39]; Li & Katikireddi [40]
Age <15 included *(n=4)*	Coombs et al. [41]; Ivey-Stephenson et al. [42]; Kapusta et al. [43]; Reccord et al. [44]
All ages/no age restriction (*n*=*5*)	Helbich et al. [45]; Bridges & Clark [46]; Micciolo et al. [47]; Chen et al. [48]; Pettrone & Curtin [49]
Book chapter *(n=1)*	Günay [50]
Only available in Spanish *(n=1)*	Chaparro-Narváez et al. [51]
Random sample *(n=1)*	Isometsä et al. [52]
Changes in rates per 100,000 *(n=1)*	Middleton et al. [53]

Table S4: Criteria for quality assessment of the selected studies

Bias Criteria
Sample representativeness:<br>0 = No information provided or specific population group (e.g., narrow age range, disease status, socioeconomic status/education selection);<br>1 = Reasonably representative of the general population, indicated by sampling method and/or provided comparison
Consistency of population in rural and urban group<br>0 = No information provided of group in rural and urban areas or population in rural and urban areas is not consistent<br>1 = Population in rural and urban group is consistent
Study period<br>0 = Study period is not clearly described or not given at all.<br>1 = Study period is described clearly.
Source of data<br>0 = Source of suicide rates is not given or source of suicide rates is not credible.<br>1 = Source of suicide rates is given and credible.
Study controls for other variables<br>0 = Study does not control for other variables.<br>1 = Study controls for age.<br>2 = Study controls for age and other factors.
Assessment of the outcome<br>0 = No reference to records is made to satisfy the requirement for confirmation of completed suicide.<br>1 = Independent or blind assessment stated in the study, or confirmation of the outcome by reference to secure records.
Presentation of the results<br>0 = Results are not clearly presented in the study or not complete.<br>1 = Results are clearly presented in the study.
Exploring trends over time<br>0 = Study did not explore trends over time.<br>1 = Study did explore trends over time.

Table S5: Newcastle-Ottawa risk of bias scores for the included studies

Author & year	Selection				Comparability	Outcome			Total score
	Representativeness of the sample population	Consistency of population in rural and urban group	Study period clear	Source of Data	Study controls for other variables	Assessment of the outcome	Presentation of the results	Exploring the trends over time	
Burrows et al. [54]	1	1	1	1	2	1	1	0	8
Caldwell et al. [55]	1	1	1	1	1	1	1	0	7
Cantor & Coory [56]	1	1	1	1	1	1	1	0	7
Carriere et al. [57]	1	1	1	1	1	1	0	1	7
Chang et al. [58]	1	1	1	1	0	1	1	1	7
Chang et al. [59]	1	1	1	1	2	1	1	0	8
Cheung et al. [60]	1	1	1	1	2	1	0	0	7
Kelleher et al. [61]	1	1	1	1	1	1	0	1	7
Levin & Leyland [62]	1	1	1	1	2	1	1	1	9
McCarthy et al. [63]	0	1	1	1	2	1	1	1	8
Morrell et al. [64]	0	1	1	1	2	1	1	0	7
Page et al. [65]	1	1	1	1	1	1	1	1	8
Park & Lester [66]	1	1	1	1	1	1	0	0	6
Pearce et al. [67]	1	1	1	1	1	1	1	1	8
Pesonen et al. [68]	0	1	1	1	2	1	0	1	7
Razvodovsky [69]	1	1	1	1	1	1	1	1	8
Sankaranarayanan et al. [70]	0	1	1	1	1	1	1	0	6
Saunderson et al. [71]	1	1	1	1	1	1	0	0	7
Shiner et al. [72]	0	1	1	1	2	1	1	1	8
Singh & Siahpush [73]	1	1	1	1	1	1	1	1	8
Sun et al. [74]	1	1	1	1	2	1	1	1	9
Taylor et al. [75]	1	1	1	1	2	1	1	0	8
Watanabe et al. [76]	0	0	1	1	2	0	0	1	5
Yip et al. [77]	1	1	1	1	1	1	1	0	7
